# Adiponectin: A New Regulator of Female Reproductive System

**DOI:** 10.1155/2018/7965071

**Published:** 2018-04-29

**Authors:** Kamil Dobrzyn, Nina Smolinska, Marta Kiezun, Karol Szeszko, Edyta Rytelewska, Katarzyna Kisielewska, Marlena Gudelska, Tadeusz Kaminski

**Affiliations:** Department of Animal Physiology, Faculty of Biology and Biotechnology, University of Warmia and Mazury in Olsztyn, Oczapowskiego 1A, 10-719 Olsztyn-Kortowo, Poland

## Abstract

Adiponectin is the hormone that belongs to the group of adipokines, chemical agents mainly derived from the white adipose tissue. The hormone plays pleiotropic roles in the organism, but the most important function of adiponectin is the control of energy metabolism. The presence of adiponectin and its receptors in the structures responsible for the regulation of female reproductive functions, such as hypothalamic-pituitary-gonadal (HPG) axis, indicates that adiponectin may be involved in the female fertility regulation. The growing body of evidence suggests also that adiponectin action is dependent on the actual and hormonal status of the animal. Present study presents the current knowledge about the presence and role of adiponectin system (adiponectin and its receptors: AdipoR1 and AdipoR2) in the ovaries, oviduct, and uterus, as well as in the hypothalamus and pituitary, the higher branches of HPG axis, involved in the female fertility regulation.

## 1. Introduction

Until the late 80s of the XX century, adipose tissue was considered only as an organ responsible for an energy storage. Since 1987, when Siiteri [1] reported that adipose tissue actively metabolizes steroid hormones, the tissue has begun to be considered as an endocrine organ and active factor in the energy metabolism regulation. For now, adipose tissue was found to be the source of a number of bioactive peptides called adipokines, which may act at both autocrine/paracrine and endocrine levels.

Adiponectin belongs to the adipokine family and initially was considered as a hormone produced exclusively by the white adipose tissue (WAT) [2–5]. A number of further studies proved that adiponectin is produced and secreted not only in the WAT but also in other tissues, like skeletal muscles, cardiomyocytes, hypothalamus, pituitary, ovaries, uterus, or placenta [6–12]. The expression of adiponectin and its receptors has been identified in the reproductive organs of many animals, including rats, mice, humans, and pigs [11–16], which indicates a potential involvement of this hormone in the reproductive system functions.

The aim of this review is to compile and systematize the current knowledge about the adiponectin system (adiponectin and adiponectin receptors) role in the structures responsible for the regulation of reproductive functions (the hypothalamus-pituitary-ovarian axis and uterus) during the reproductive cycle and early gestation.

### 1.1. The Hormone

Adiponectin is a 244-amino-acid protein with molecular weight of 30 kDa. The hormone contains four domains: the amino-terminal signal sequence, a nonconserved variable region, a collagenous domain, and a carboxy-terminal globular domain [3]. Adiponectin circulates in the serum in three main homomultimer fractions: trimer (low molecular weight, LMW), hexamer (medium molecular weight, MMW), and multimer, containing 12 to 18 adiponectin molecules (high molecular weight, HMW) [4]. Fruebis et al. [17] identified the fourth fraction of the adipokine, globular adiponectin, which is formed by the proteolytic cleavage of full-length hormone. In the serum, adiponectin occurs at approximately 0.01% of total plasma proteins, at the *μ*g/ml concentrations [18]. Concentration of this adipokine in the plasma estimates from 3 to 30 *μ*g/ml in humans, 2 to 7 *μ*g/ml in rats, and 3 to 4 *μ*g/ml in pigs [18–20]. Despite the fact that adiponectin is produced mainly by the WAT, its serum concentration is reversely correlated with the body mass index and the overall mass of WAT [18]. Adiponectin plasma concentration was found to be sex dependent. Sexual dimorphism of the hormone serum concentration was confirmed in human and mice [21, 22]. Physiological concentrations of the adipokine were higher in women (12.5 ± 0.3 *μ*g/ml) than in men (8.7 ± 0.3 *μ*g/ml) and prepubertal individuals. For more, in pubescent boys, adiponectin concentrations were significantly lower (5.6 ± 0.5 *μ*g/ml) than in girls of the same age (7.1 ± 0.5 *μ*g/ml), which suggests that adiponectin serum concentration may be dependent on the androgen concentration [22–24].

Adiponectin exhibits pleiotropic properties. The hormone is known for its involvement in the control of metabolism and insulin sensitisation. In the liver, adiponectin promotes glucose transport, inhibits gluconeogenesis, activates oxidation of fatty acids, and enhances insulin sensitivity promoting phosphorylation of the insulin receptor [25, 26]. In WAT, adiponectin promotes basal glucose uptake, insulin-stimulated glucose uptake, and regulates fat lipid metabolism inhibiting lipolysis [25, 27]. Adiponectin is also known for its anti-inflammatory properties. The adipokine may attenuate inflammation processes in different types of tissues, like endothelial cells, muscle, epithelial cells, and macrophages [28–30]. Its antiatherogenic and anticancerogenic properties have also been proven [31–34].

Expression of adiponectin gene and protein was confirmed in the reproductive system. Adiponectin expression was noted, among others, in the human endometrium as well as uterus, trophoblasts, and conceptuses of mice and pigs [12, 14, 16]. For more, in pig the concentration of adiponectin in plasma, as well as, the expression of the hormone and its receptors in the ovary and uterus were found to be dependent on the phase of the oestrous cycle or the stage of gestation. In the porcine ovaries, adiponectin expression on both gene and protein expression levels was found to be enhanced during the luteal phase of the oestrous cycle, when compared to the follicular phase of the cycle. In the porcine uterus, the highest expression of the adiponectin gene was observed on days 14 to 16 and 2 to 3 of the oestrous cycle, in the endometrium and myometrium, respectively, whereas during the early gestation period, on days 15 to 16 of gestation in both the endometrium and myometrium. During the oestrous cycle, the hormone concentration in the blood plasma was constant during the luteal phase (from days 2 to 3 to days 14 to 16) and decreased during the follicular phase (days 17 to 19 of the cycle). During the early gestation period, the highest concentration of adiponectin in the porcine plasma was observed on days 15 to 16 of gestation, whereas the lowest on days 30 to 32 of pregnancy. Taken together, data presented above indicates that adiponectin actions may be dependent on the actual hormonal status of animals [15, 16, 20, 35, 36]. The above also suggests that adiponectin may be involved in the regulation of the reproductive functions.

### 1.2. Adiponectin Receptors

In the organism, adiponectin actions are mediated *via* two distinct receptors: adiponectin receptor type 1 (AdipoR1) and adiponectin receptor type 2 (AdipoR2). Mouse AdipoR1 and AdipoR2 share 66.7% homology in its amino acid sequence. Both receptors are integral membrane proteins consisting of seven transmembrane domains, which make them similar to the G-protein-coupled receptors family. However, the N-terminus of the proteins is located internally and the C-terminus externally, which is opposite to the topology of G-protein-coupled receptors [37]. Human AdipoR1 protein consists of 375-amino-acids and has a molecular weight of 42.4 kDa. Human, mouse, and porcine AdipoR1 gene are located on the chromosome 1p36.13-q41, 1 E4, and 10p11, respectively. The receptor has a greater affinity to the trimers and globular domain of adiponectin and is mostly expressed in the skeletal muscles [37–39]. AdipoR1 acts *via* AMP kinase and mitogen-activated protein kinase [40]. Human AdipoR2 protein consists of 386-amino-acids with a molecular weight of 48.3 kDa. Its gene is located on the chromosome 12p13.31, 6 F1, and 5q25 for human, mouse, and pig, respectively. AdipoR2 has a higher affinity for the multimeric forms of adiponectin and is highly expressed in the liver [37–39]. The receptor acts primarily through the peroxisome proliferator-activated receptor *α* (PPAR*α*) pathway [39]. Hug et al. [41] reported the existence of third adiponectin receptor, T-cadherin. The protein is expressed mainly in the vascular endothelial cells and smooth muscles. T-cadherin binds MMW and HMW adiponectin; however, it has no influence on adiponectin cell signaling since its protein has no intracellular domain. It is hypothesized that T-cadherin acts only as an adiponectin binding protein [38, 41–43].

Similar to adiponectin, AdipoRs' (AdipoR1 and AdipoR2) were found to be expressed in many tissues, including the structures responsible for the reproduction. AdipoRs' expression was confirmed in all structures of the hypothalamic-pituitary-ovarian axis (HPG axis). The presence of adiponectin receptors was observed in human, rodent and porcine hypothalami [44–46], human, rat and porcine pituitaries [9, 47, 48], and rat and porcine ovarian follicles, and corpora lutea (CL) [20, 48]. Moreover, AdipoRs' expression was observed also in the human endometrium and uteri and conceptuses of mice and pigs [12, 14, 16].

## 2. Adiponectin and H-P-G Axis

### 2.1. Hypothalamus

**Figure 1:**
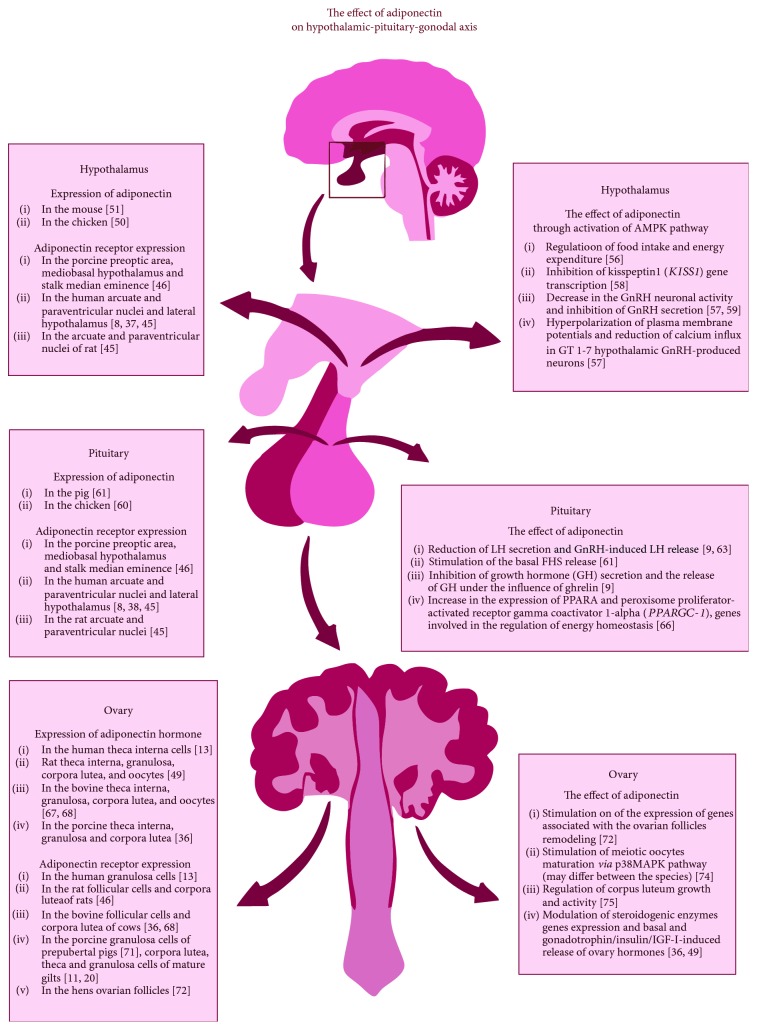
The evolvement of adiponectin system in the regulation of hypothalamic-pituitary-gonadal axis (H-P-G axis) in different animal species. The left side of the figure presents the expression of adiponectin and adiponectin receptors in the particular tissues of the H-P-G axis. The right side of the figure presents the effect of the hormone on the target tissues.

Adiponectin receptor expression in the hypothalamus was confirmed in many species, including humans, rodents, and pigs [44–46]. In the hypothalamus, AdipoRs' were expressed in the porcine preoptic area, mediobasal hypothalamus and stalk median eminence, human and rat arcuate and paraventricular nuclei, and human lateral hypothalamus [8, 37, 44–46]. Expression of adiponectin hormone was confirmed in the murine and chicken brains; however, no immunopositive cells for adiponectin were observed in the human hypothalamus or infundibular stalk [8, 49, 50] (Figure 1). Adiponectin may be also supplied by the blood. The hormone has been detected in human, mice, and rat cerebrospinal fluid (CSF) [44, 51–54]. The hormone concentrations in the CSF are many times lower (0.1%), when compared to blood plasma. In the CSF, adiponectin occurs only in the LMW and MMW forms, with the dominant contribution of the LMW form, which suggest an inability of high-molecular complexes to cross the blood-brain barrier [52].

The pleiotropic effect of adiponectin in the mouse hypothalamus has been observed. The hormone, *via* AdipoR1, enhanced AMPK activity in the arcuate hypothalamus, which resulted in the stimulation of food intake and decreased energy expenditure. Moreover, in the adiponectin-deficient mice, the AMPK phosphorylation was decreased, which caused an increase in the energy expenditure and decreased food intake [55]. The second function of adiponectin in the hypothalamus is the hormone involvement in the regulation of gonadoliberin (GnRH) secretion. Adiponectin, *via* activation of the AMPK, inhibited GnRH secretion and caused a hyperpolarization of plasma membrane potentials and reduction of calcium influx in GT1-7 mouse hypothalamic GnRH-produced neurons [56]. For more, adiponectin, also through AMPK pathway, inhibits the gene transcription of kisspeptin 1 *(KISS1)*, the upstream signal for GnRH release [57]. Klenke et al. [58] reported that mouse GnRH neurons express AdipoR2 and adiponectin may decrease the GnRH neuronal activity rapidly *via* the AMPK pathway. The above findings indicate the potential role of adiponectin as a metabolic regulator of the reproductive functions *via* its influence on GnRH release (Figure 1).

### 2.2. Pituitary

The expression of adiponectin mRNA in the pituitary gland was described in a number of species [9, 59–61] (Figure 1). In humans, adiponectin expression was localized mainly in growth hormone (GH), follicle-stimulating hormone (FSH), luteinizing hormone (LH), and thyroid-stimulating hormone- (TSH-) producing cells [8]. The expression of adiponectin receptors in the pituitary was also confirmed in many species including humans, rats, and pigs [8, 9, 47, 48]. Psilopanagioti et al. [8] localized AdipoRs' expression in the human gonadotrophs, somatotrophs, and thyrotrophs, but not in corticotrophs or lactotrophs. The expression of AdipoRs' in the pituitary suggests that the hormone may regulate central endocrine axes and participate in the control of metabolic homeostasis.

Rodriguez-Pacheco et al. [9] proposed that locally produced adiponectin may affect pituitary hormone secretion. The presence of this adipokine in the rat and mouse pituitary cell cultures resulted in the reduction of LH secretion and LH release induced by GnRH [9, 62]. For more, in rats, adiponectin has been observed to inhibit growth hormone (GH) secretion and the release of GH under the influence of ghrelin [9]. Adiponectin had also a stimulatory effect on ACTH secretion in the primary culture of pituitary cells *via* an AMPK-dependent mechanism in the rat's pituitary corticotroph cells [63]. In the *in vitro* studies on the porcine primary pituitary cells, adiponectin stimulated the basal FSH release. Administration of the hormone affected GnRH- and insulin-induced LH and FSH secretion dependently on the phase of the oestrous cycle [60]. As mentioned in the previous subsection, adiponectin had an inhibitory effect on the GnRH release [56]. However, GnRH suppressed adiponectin expression in the rat primary pituitary cell culture as well as in mouse L*β*T2 gonadotroph cell line. The inhibitory action of GnRH was mediated *via* the calcium and protein kinase A intracellular pathways. Interestingly, GnRH did not affect the expression of both AdipoRs' [61]. As we observed in our unpublished studies (Szeszko et al., unpublished data), adiponectin affects the global gene expression in the porcine primary pituitary cell culture. Results of the studies show that adiponectin influences the group of genes responsible for MAPK cascade, which plays a key role in the transduction of extracellular signals to cellular responses and regulates the expression of several gene encoding gonadotroph functions. The activation of MAPK ERK1/2 signaling pathway is necessary for, among others, steroidogenesis and steroidogenic gene expression in granulosa cells [64]. For more, adiponectin increased the expression of PPARA and peroxisome proliferator-activated receptor gamma coactivator 1-alpha (*PPARGC-1*) genes involved in the regulation of energy homeostasis, especially fatty acid oxidation and carbohydrate metabolism (Figure 1). Wang et al. [65] hypothesize that adiponectin may act as a mediator of nutrition and reproduction in sheep. Fasting of prepubertal eves increased serum adiponectin concentrations and enhanced *AdipoR1* and *AdipoR2* gene expression, which showed the negative correlation with the LH *β*-subunit and FSH *β*-subunit gene expression.

The presence of adiponectin system in the pituitary, especially in the gonadotroph cells, and its influence on the LH and FSH release, *via* the modulation of the AMPK and MAPK signaling pathways, indicates the important role of this hormone in the regulation of the reproductive functions at the higher branches of HPG axis, in response to the actual metabolic status of female.

### 2.3. Ovaries

To assent adiponectin as a key factor involved in the reproductive system regulation, it is important to investigate its direct role in gonads. The expression of adiponectin on both gene and protein level was noted in the women theca interna cells [13], theca interna, granulosa, corpora lutea, and oocytes of rats [66] and cows [67, 68], in theca interna, granulosa, and corpora lutea of pigs [36] (Figure 1). The concentration of this adipokine was measured in human (2.209 ± 0.85 *μ*g/ml) and cattle (19.4 ± 1.4 *μ*g/ml) follicular fluid [69, 70]. In turn, the AdipoRs' expression was reported in the human granulosa cells [13], follicular cells, and corpora lutea of rats [46] and cows [36, 68], in granulosa cells of prepubertal pigs [71], corpora lutea, theca and granulosa cells of mature gilts [11, 20], and in the ovarian follicles of hens (mRNA only) [72].

It has been postulated that adiponectin may take part in the initiation of preovulatory changes in the ovary and modulate the ovarian steroidogenesis process. The presence of adiponectin in the primary granulosa cell culture from prepubertal gilts stimulated the expression of genes associated with the remodeling of the ovarian follicles, including cyclooxygenase-2, prostaglandin E synthase, and vascular endothelial growth factor genes [72]. In pigs, adiponectin modulates steroidogenic enzymes and steroidogenic acute regulatory protein (*StAR*) gene expression, increasing *StAR* transcript abundance and reducing the cytochrome P450 aromatase (*CYP19A3*). It has been observed that the adipokine affects basal and gonadotrophin-/insulin-induced release of progesterone (P_4_), oestradiol (E_2_), and testosterone by the porcine luteal and follicular cells, respectively [36]. In the rat primary granulosa cells, adiponectin had no effect on the basal steroid secretion but caused an increase in P_4_ and E_2_ production, when combined with the insulin-like growth factor-I (IGF-I) [66]. In the *in vitro* studies on bovine ovaries, Lagaly et al. [71] observed that adiponectin decreased insulin-induced P_4_ and androstenedione (A_4_) production, inhibited IGF-I, as well as induced *LH receptor*, P450 side-chain cleavage enzyme *(CYP11A1)*, and cytochrome P450c17 *(CYP17A1)* gene expression in theca cells, and decreased *LH receptor* gene expression in granulosa cells. For more, adiponectin may also take part in the oocyte maturation process. AdipoR1 and AdipoR2 were found to be expressed in the porcine oocytes and cumulus cells in both small and large follicles, and adiponectin was found to stimulate meiotic maturation of oocytes derived from large follicles *via* p38MAPK pathway [73]. On the other hand, in the bovine *in vitro* studies, adiponectin did not affect oocyte maturation [68], which implies that adiponectin role in the oocyte maturation may differ between the species. The microarray analysis of Szeszko et al. [74] indicates the modulatory effect of adiponectin on the porcine ovarian cells during the luteal phase of the oestrous cycle. The researchers observed that adiponectin influences the number of genes, including steroidogenic enzymes, genes responsible for prostaglandin synthesis, or genes responsible for vascularization, which suggests the important role of this adipokine in the regulation of CL growth and activity (Figure 1).

Disorders in the adiponectin serum concentrations and its influence on the ovarian steroidogenesis have been linked with the polycystic ovary syndrome (PCOS). PCOS is one of the commonest endocrine disorders in women, and hyperandrogenism is one of its hallmarks. Theca cells are recognized as one of the primary sources of excess androgen biosynthesis in women with PCOS [75]. In the PCOS women, the concentration of adiponectin in the blood plasma was 16 *μ*g/ml which is about 23.5% less than in the plasma of healthy individuals (about 20 *μ*g/ml) [76]. Another characteristic issue for PCOS is the difference in the adiponectin multimer ratio and concentrations, when compared to the control group. Aroda et al. [77] reported lower HMW adiponectin serum levels in women with PCOS, whereas O'Connor et al. [78] observed selectively reduced HMW fraction in PCOS-positive individuals, independently to the body mass index. In the bovine theca cells, adiponectin suppressed A_4_ production and gene expression of key enzymes in the androgen synthesis pathway. In turn, the knockout of AdipoRs' genes resulted in an increase in the A_4_ secretion by the bovine theca cells. For more, in women polycystic ovaries, a significantly lower proportion of theca cells expressing AdipoR1 and AdipoR2 was observed, when compared to the normal ovaries [79]. Also in the mouse ovary, adiponectin reduced A_4_ secretion and oxidative stress protein concentrations, which may be potentially linked with the pathogenesis of PCOS associated to obesity [80].

The presence of adiponectin and its receptors in the ovaries during all periods of the oestrous cycle, taken together with the data presented above, indicates the important role of this adipokine in the regulation of oocyte maturation, CL formation and activity, and a proper course of the oestrous/menstrual cycle *via* its influence on the steroid production process. A clear confirmation of this statement may be a link between adiponectin and its receptor concentrations and the PCOS disorder.

## 3. Reproductive Tract

**Figure 2:**
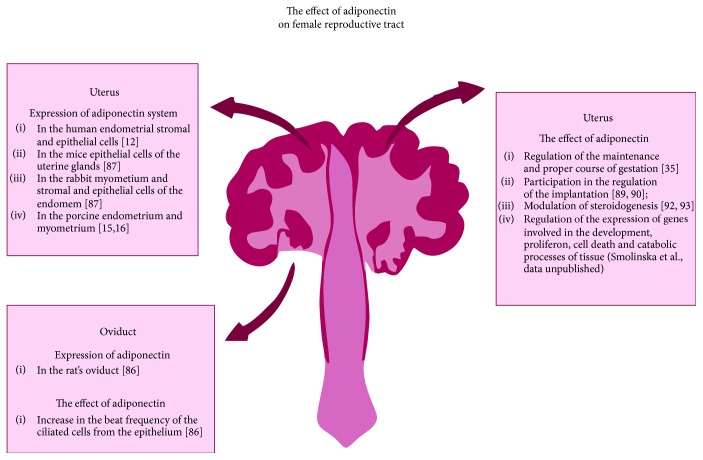
The effect of adiponectin on the female reproductive tract. The figure presents the expression of the adiponectin and adiponectin receptors, as well as the hormone action in the oviduct and uterus.

Adiponectin system is supposed to influence reproductive functions not only at the central level *via* the H-P-G axis regulation but also locally *via* the hormone actions across the reproductive tract. In mammals, the oviduct, besides its obvious role in the oocyte and embryo transport, is an important source of hundreds of macromolecules derived by oviduct epithelium, such as enzymes, protease inhibitors, growth factors, and a group of oviductins [81–83]. The expression of adiponectin gene and protein in the rat oviduct has been confirmed by Archanco et al. [84]. In the oviductal secretory epithelial cells of cyclic rats, adiponectin expression was changing throughout the oestrous cycle, increasing from proestrous to oestrous. Oses et al. [85] indicated that in the *in vitro* primary cultures of ciliated cells from the rat epithelium adiponectin treatment resulted in an increased ciliary beat frequency (Figure 2). In the uterus, the expression of the adiponectin system was reported, among others, in human endometrial stromal and epithelial cells, mouse epithelial cells of the uterine glands, rabbit myometrium, and endometrial stromal and epithelial cells, as well as in porcine endometrium, myometrium, and trophoblasts [12, 15, 16, 86] (Figure 2). In the porcine uterine luminal fluid, adiponectin concentration varies between the oestrous cycle (4.7 *μ*g/ml) and early pregnancy period (8.7 *μ*g/ml), which taken together with the data presented above suggests the important role of adiponectin in the maintenance and proper course of gestation [35]. Adiponectin system has been suggested to play an important role during early pregnancy period, especially implantation. Adiponectin receptor expression was found to be increased in the primary cultures of human stromal cells during the decidualization process, what may be an evidence for the modulatory influence of adiponectin on the uterine receptivity during pregnancy [87]. Expression of adiponectin, as well as AdipoRs', was found in both rabbit and mouse trophoblasts and embryoblasts, suggesting that during the pre- and peri-implantation period, adiponectin may be involved in the crosstalk between mother and embryo [86]. Another evidence for the regulatory function of adiponectin during early gestation was provided by Dos Santos et al. [88], who indicated the lower expression of adiponectin receptor genes in the uteri of women with iterative implantation failures. For more, in women with endometriosis, in which high rate of implantation failure was observed, adiponectin serum level was lower (13.1 *μ*g/ml), when compared to the healthy individuals (15.9 *μ*g/ml) [89].

As described in the above subsection, it has been proven that adiponectin exerts a modulatory influence on the steroidogenesis process in the ovary. In 2008, Franczak and Kotwica [90] indicated the steroidogenic activity of the porcine endometrium and myometrium and indicated that the uterus is an alternative source of steroid hormones. Smolinska et al. [91] proved that adiponectin may modulate not only ovarian but also endometrial and myometrial steroidogenesis. In the *in vitro* studies on porcine uterine tissues, adiponectin modulated the gene expression of key enzymes involved in the steroid synthesis: *StAR*, *CYP11A1*, and *HSD3B1* (3*β*-hydroxysteroid dehydrogenase), as well as influenced the secretion of P_4_ and A_4_ by the tissues. For more, adiponectin has been found to modulate also the prostaglandin synthesis pathway in the porcine uterus. On day 15 of pregnancy, the presence of adiponectin and insulin in the *in vitro* cultures of porcine endometrial cells enhanced the expression of gene encoding enzymes important for the prostaglandin synthesis, such as cyclooxygenase 2, as well as vascular endothelial growth factor and peroxisome proliferator-activated receptor gamma gene expression [92]. Our unpublished data, concerning the influence of adiponectin on the global gene expression in the porcine endometrium during early pregnancy indicates that the hormone is involved in the regulation of a number of processes important for the tissue growth and development. We found that adiponectin provoked an increase in the group of genes responsible for development and proliferation and suppressed the expression of genes connected with the cell death and catabolic processes. For more, the adipokine modulated the expression of genes involved in the steroid and prostaglandin synthesis, or metabolism, which confirmed the previous results and expanded our knowledge about adiponectin's interaction networks in the pregnant endometrium (Smolinska et al., data unpublished) (Figure 2).

Adiponectin and adiponectin receptor genes and protein expression in the porcine uterus, as well as adiponectin concentration in the porcine uterine luminal fluid, change during both the oestrous cycle and early pregnancy period, which suggests that adiponectin system expression in the uterus strongly depends on the animals hormonal status [15, 16, 35]. Those speculations were confirmed by the *in vitro* studies of Dobrzyn et al. [93–95], which indicated the modulatory effect of steroid hormones, P_4_, oestrone, and E_2_, as well as prostaglandins: E_2_ and F_2*α*_ on the adiponectin system expression in the porcine pregnant uterus. The effect of the steroids and prostaglandins on the adiponectin system expression in the endometrium and myometrium was found to be tissue specific and dependent largely on the period of early gestation. The modulatory effect of steroid hormones on the adiponectin system was confirmed also in the cultures of human endometrial stromal and epithelial cell lines, in which E_2_ and P_4_ stimulated the expression of both AdipoRs' genes during the decidualization process [87].

Taken together, the data presented above indicates that adiponectin act as an important hormonal regulator in a number of processes occurring in the reproductive tract during both the oestrous cycle and early pregnancy, *via* its influence on, among others, steroid hormones and prostaglandin synthesis by the tissues.

## 4. Embryos and Placenta

Adiponectin system is suspected to take part in the maternal-foetus interactions. The elements of adiponectin system were found in early developing embryos of pig [16, 73], mouse embryos at all stages of preimplantation development [14], and rabbit and mouse-implanting blastocysts [86]. It was shown that adiponectin may promote embryo development to blastocyst stage in the porcine *in vitro* cultures [73]. For more, it was proven that adiponectin treatment resulted in doubling of mouse blastocyst formation, when compared to the control group, which indicates the regulatory role of the hormone in cell proliferation during the embryo development. In the mice *in vitro* blastocyst culture, the influence of adiponectin resulted in an increased proportion of embryos with high cell numbers, indicating that adiponectin may affect the development of the preimplantation embryo [96]. It is hypothesized that in early developing embryos, adiponectin, via AdipoRs', may downstream fatty acid oxidation and replete energy stores in the developing embryo before the implantation [14]. What is more, in rabbit blastocysts, adiponectin was found to enhance PRKA alpha 1/2 (PRKAA1/2) phosphorylation and decrease the expression of key regulator of gluconeogenesis, the phosphoenolpyruvate carboxykinase 2 (PCK2). It was indicated that *via* the phosphorylation of PRKAA1/2, adiponectin influences the glucose metabolism of blastocyst, which results in a decrease of gluconeogenesis and an increase in glycolysis [97].

**Figure 3:**
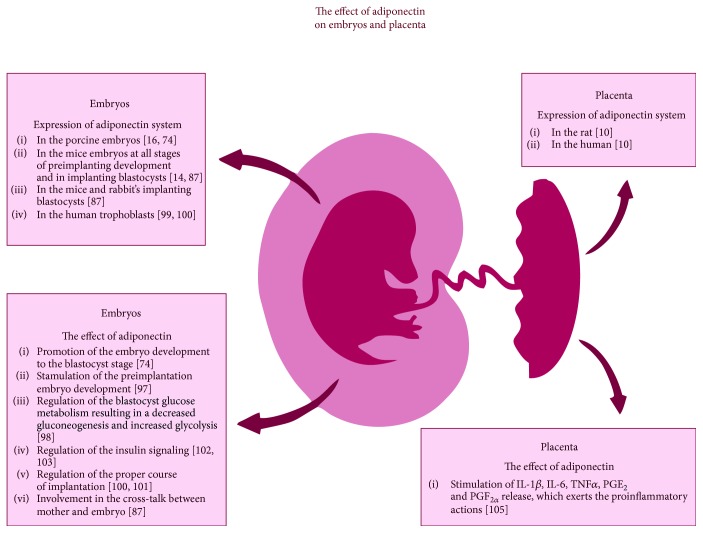
The effect of adiponectin on the embryos and placenta. The left side of the figure presents the expression of adiponectin system, as well as adiponectin effect on the embryos of different mammalian species. The right side of the figure presents the expression of adiponectin and its receptors and the hormone action in the mammalian placenta.

Adiponectin system expression was confirmed also in the porcine and human trophoblasts [16, 98, 99]. However, human trophoblast cells expressed only AdipoRs' [98, 99] (Figure 3). Adiponectin was found to act stimulatory on the trophoblast cell migration and invasion in the *in vitro* culture of human HTR-8/SVneo cell lines, which suggests that adiponectin may be a positive regulator of the early invasion process [99]. On the other hand, the adipokine was also found to exert an antiproliferative effect on human trophoblastic JEG-3 and BeWo choriocarcinoma cell lines, which points out that the hormone may act also as a regulator of trophoblastic cell proliferation and, in consequence, on the proper course of implantation [100]. Adiponectin was also found to attenuate insulin signaling in the primary human trophoblast cells, which, in result, inhibited insulin-stimulated amino acid transport. Those findings may have important implications in the pregnancy disorders linked with altered maternal adiponectin levels [101, 102] (Figure 3).

The gene expression of adiponectin and its receptors was described in the rat and human placenta [10]. Similar to the trophoblast, also in the placenta, adiponectin is supposed to play a role in adapting energy metabolism at the maternal-fetal interface. In the human placenta, adiponectin system was found to be regulated by the cytokines (including TNF*α*, IFN-*γ*, and IL-6) and leptin [103]. Adiponectin was also found to enhance the release of IL-1*β*, IL-6, TNF*α*, PGE_2_, and PGF_2*α*_, exerting proinflammatory actions in human placenta [104] (Figure 3). The above findings suggest the existence of autoregulatory loop between proinflammatory cytokines and adiponectin in the placenta, which may be important for the proper growth and functioning of this organ.

## 5. Conclusion

Adiponectin, the adipocyte-derived hormone is an important factor taking part in the regulation of organism energy metabolism. Herein, the authors gathered available data concerning the inherence of adiponectin system in the structures responsible for the female reproductive system functioning and proving the regulatory role of adiponectin in these organs. Data presented above indicate that adiponectin is an important factor regulating the reproductive functions dependently on the actual metabolic status of animal and *vice versa*, regulating female metabolism according to the hormonal status of animal during both menstrual/oestrous cycle and pregnancy.
